# Use of drug therapy in the management of symptomatic ureteric stones in hospitalized adults (SUSPEND), a multicentre, placebo-controlled, randomized trial of a calcium-channel blocker (nifedipine) and an α-blocker (tamsulosin): study protocol for a randomized controlled trial

**DOI:** 10.1186/1745-6215-15-238

**Published:** 2014-06-20

**Authors:** Sam McClinton, Kathryn Starr, Ruth Thomas, Graeme McLennan, Gladys McPherson, Alison McDonald, Thomas Lam, James N’Dow, Mary Kilonzo, Robert Pickard, Ken Anson, Jennifer Burr

**Affiliations:** 1 Aberdeen Royal Infirmary, Foresterhill, Aberdeen AB25 2ZN, Scotland; 2 Academic Urology Unit, University of Aberdeen, Health Sciences Building, Foresterhill, Aberdeen AB25 2ZD, Scotland; 3 Health Services Research Unit, University of Aberdeen, Health Sciences Building, Foresterhill, Aberdeen AB25 2ZD, Scotland; 4 Health Economics Research Unit, University of Aberdeen, Polwarth Building, Foresterhill, Aberdeen AB25 2ZD, Scotland; 5 Institute of Cellular Medicine, Newcastle University, Newcastle Upon Tyne NE2 4HH, England; 6 St George's Hospital, London SW17 0QT, England; 7 Department of Medical & Biological Sciences, University of St Andrews, St Andrews KY16 9TF, Scotland

**Keywords:** medical expulsive therapy, nifedipine, tamsulosin, ureteric stone

## Abstract

**Background:**

Urinary stone disease is common, with an estimated prevalence among the general population of 2% to 3%. Ureteric stones can cause severe pain and have a significant impact on quality of life, accounting for over 15,000 hospital admissions in England annually. Uncomplicated cases of smaller stones in the lower ureter are traditionally treated expectantly. Those who fail standard care or develop complications undergo active treatment, such as extracorporeal shock wave lithotripsy or ureteroscopy with stone retrieval. Such interventions are expensive, require urological expertise and carry a risk of complications.

Growing understanding of ureteric function and pathophysiology has led to the hypothesis that drugs causing relaxation of ureteric smooth muscle, such as the selective α-blocker tamsulosin and the calcium-channel blocker nifedipine, can enhance the spontaneous passage of ureteric stones. The use of drugs in augmenting stone passage, reducing the morbidity and costs associated with ureteric stone disease, is promising. However, the majority of clinical trials conducted to date have been small, poor to moderate quality and lacking in comprehensive economic evaluation.

This trial aims to determine the clinical and cost-effectiveness of tamsulosin and nifedipine in the management of symptomatic urinary stones.

**Methods/design:**

The SUSPEND (Spontaneous Urinary Stone Passage ENabled by Drugs) trial is a multicentre, double-blind, randomized controlled trial evaluating two medical expulsive therapy strategies (nifedipine or tamsulosin) versus placebo.

Patients aged 18 to 65 with a ureteric stone confirmed by non-contrast computed tomography of the kidney, ureter and bladder will be randomized to receive nifedipine, tamsulosin or placebo (400 participants per arm) for a maximum of 28 days. The primary clinical outcome is spontaneous passage of ureteric stones at 4 weeks (defined as no further intervention required to facilitate stone passage). The primary economic outcome is a reduction in the incremental cost per quality-adjusted life years, determined at 12 weeks. The analysis will be based on all participants as randomized (intention to treat). The trial has 90% power with a type I error rate of 5% to detect a 10% increase in primary outcome between the tamsulosin and nifedipine treatment groups.

**Trial registration:**

ISRCTN69423238; EudraCT number: 2010-019469-26

## Background

Urinary stone disease is very common, with an estimated prevalence among the general population of 2% to 3% and an estimated lifetime risk of 1 in 8 for white males [1] and 5% to 6% for white females [2], with men forming stones three times as often as women. Urinary stones often recur and the lifetime recurrence rate is approximately 50% [3]. The interval between recurrences is variable, with approximately 10% within 1 year, 35% within 5 years and 50% within 10 years [2]. The increased incidence of urinary stones in the industrialized world is associated with improved standards of living (mainly owing to the high dietary intake of proteins and minerals) and there is also an association with ethnicity and region of residence [4]. All urinary tract stones, and ureteric stones in particular, have a significant impact on patients’ quality of life. They are a common cause of emergency hospital admission due to severe pain with over 15,000 hospital admissions in England annually [5] using over 21,500 bed days. The pain leads to a requirement for analgesia, time off work and, often, repeated hospital admissions for therapeutic interventions.

A clinical guideline on the management of ureteric stones by the European Association of Urology and the American Urological Association [6] estimates that 68% of stones ≤5 mm and 47% of stones 5 to 10 mm in size can be expected to pass spontaneously and concluded that the majority of these stones pass within 4 to 6 weeks of presentation. Stones in the distal ureter pass more readily than stones located more proximally. The majority of the studies included in the guideline meta-analysis assessed stones in the distal (lower) ureter only. Consequently, patients with favourable features and with smaller stones in the lower ureter are traditionally treated expectantly. Those who fail standard supportive care (which involves analgesia, anti-emetics if nauseated, and intravenous fluids if there is associated vomiting), or who subsequently develop complications, undergo active treatment, such as extracorporeal shock wave lithotripsy, ureteric stenting, ureteroscopy with stone retrieval or *in-situ* lithotripsy, or percutaneous nephrostomy insertion. However, such interventions are expensive, require urological expertise and carry a risk of complications. For instance, extracorporeal shock wave lithotripsy is associated with up to 5% risk of sepsis and up to 8% risk of impaction of stone fragments causing urinary obstruction (‘Steinstrasse’), whilst ureteroscopy is associated with up to 4% risk of sepsis and up to 6% risk of ureteric injury [6].

In recent years, a growing understanding of ureteric function and pathophysiology has led to the hypothesis that drugs that cause relaxation of ureteric smooth muscle can enhance the spontaneous passage of ureteric stones [7-9]. The selective α-blocker, tamsulosin has specificity for α-1A and α-1D receptor subtypes [10,11], whilst other α-blockers variably block all α-1 receptor subtypes in a non-specific manner [12-14]. Similarly, calcium-channel blockers, such as nifedipine, inhibit ureteric smooth muscle contraction [15,16]. The use of both classes of drugs in augmenting the passage of ureteric stones has been termed medical expulsive therapy (MET) and this is proposed as a way to enhance stone passage and avoid the need for further intervention.

Two recent meta-analyses have reported the potential role of α-blockers and calcium-channel blockers in MET. Hollingsworth and co-workers [17] included nine randomized controlled trials, which included 693 subjects, although all but one trial had serious methodological flaws. Studied interventions included the calcium-channel blocker nifedipine and several different α-blockers whilst the comparative control arms included placebo, other vasodilators, antispasmolytics, anticholinergic therapy and corticosteroids. Overall spontaneous stone passage occurred in 47% of the control group whilst patients given MET with either drug were 65% more likely to pass the stone, with an absolute risk reduction of 31%. Three studies reported a head-to-head comparison between nifedipine and α-blockers. Two of these studies did not report any statistically significant difference in stone passage rates between the two drugs, whilst one study found the α-blocker to be superior to nifedipine, with a relative risk reduction of 26%.

In a more recent systematic review and meta-analysis, Singh and colleagues [18] included 22 studies; of which 13 assessed α-blockers, 6 assessed nifedipine, and 3 assessed both drugs against control. In the pooled analysis of 16 studies using α-blockers (*n* = 1,235), those receiving active treatment were 59% more likely to pass the stone, with a baseline stone passage rate of 50% in the control group. The incidence of mild adverse effects was 4%. The corresponding pooled result for nifedipine (nine studies, *n* = 686) showed that active treatment gave a 50% increased likelihood of stone passage, with an absolute risk reduction of 26%. The incidence of mild adverse effects was 15%. Both drugs significantly shortened, by between 2 and 6 days, the average time to stone expulsion [18]. However, the overall quality of the trials was poor.

In both meta-analyses, the majority of studies involved stones <10 mm located in the lower (distal) ureter. These two reviews both concluded that a large, high-quality randomized controlled trial is required to confirm their findings, suggesting that MET with either drug class can enhance spontaneous stone passage rate. In addition, several studies have previously reported that MET can significantly reduce the pain burden among patients in terms of reducing the frequency of pain episodes, pain severity and analgesic requirements.

However, more recent results provided by Bensalah and co-workers [19] appear to challenge the notion that α-blockers enhance spontaneous ureteric stone passage. The study, recently presented as an abstract, was a prospective, multicentre, randomized, double-blind, placebo-controlled trial, which evaluated the efficacy of tamsulosin versus placebo in patients with ureteric colic caused by distal ureteric stones. A total of 129 patients were treated for 42 days or until stone expulsion. At 42 days, there was no significant difference between the spontaneous expulsion rates between placebo (70.5%) and tamsulosin (77.0%; *P* = 0.41), nor in the mean stone passage times (10.1 and 9.6 days, respectively). Nevertheless, the overall mean stone diameter was 3.1 mm, which is smaller than all of the earlier studies included in the meta-analyses by Hollingsworth [17] and Singh [18]. The spontaneous stone passage rate in the placebo arm was high (70.5%) in comparison with other studies included in the two meta-analyses.

There is limited evidence on the cost-effectiveness of MET; an indirect cost-benefit analysis based on cost data from the USA and four European countries suggested that the use of tamsulosin could potentially result in a cost saving of US$ 1,132 per patient episode over conventional ‘watchful waiting’ [20].

In summary, the role of MET in reducing the morbidity and economic costs associated with ureteric stone disease is promising. The majority of clinical trials conducted to date have been small and of poor to moderate quality in terms of trial methodology or design. Furthermore, they have lacked a comprehensive economic evaluation. There is thus an urgent need for a definitive randomized controlled trial, such as that described in this protocol to inform the clinical management of patients with ureteric stone disease.

For the purposes of this randomized controlled trial, we have chosen to compare tamsulosin versus nifedipine. The weight of available evidence supports the use of tamsulosin as the α-blocker of choice in MET for ureteric stones. In the two previous reviews [17,18], tamsulosin was the agent of choice in 13 out of 16 randomized controlled trials. As discussed earlier, there also appears to be a theoretical advantage in using tamsulosin, owing to its specificity for the α-1A and α-1D adrenergic receptor subtypes. Similarly, the reviews also suggest that nifedipine should be the calcium-channel blocker of choice. The eight randomized controlled trials identified in Singh [17] and Hollingsworth [18] that examined the efficacy of calcium-channel blockers all used nifedipine. Nifedipine is also in widespread use in the UK’s National Health Service (NHS) for other indications.

The main anticipated risk to participants is that they suffer an adverse reaction to the trial medication. Treatment with α-blocker or nifedipine is associated with a small risk of adverse effects. In the report by Singh and colleagues [18], the incidence of mild adverse effects was 4% with α-blocker and 15% with nifedipine. However, both trial drugs are in common use for different indications, and the undesirable effects (such as postural hypotension and tachycardia) are well recognized. Patients with a contraindication to either drug will not be included in the trial. The off-label use of tamsulosin in women is well documented in the literature and there have not been any reports of any specific adverse reactions to treatment in female participants. However, the risks of tamsulosin use during pregnancy are unknown and nifedipine is contraindicated during pregnancy. Two suitable ‘highly effective’ forms of contraception must be used by women of childbearing potential entering the trial.

The aim of this trial is to determine the clinical effectiveness and cost-effectiveness of the use of tamsulosin and nifedipine in the management of symptomatic urinary stones. The potential benefits to participants are that the pain and discomfort caused by their ureteric stones will be relieved sooner and the avoidance of additional treatment (such as ureteroscopy or extracorporeal shock wave lithotripsy) will be reduced by 25% to 45%.

In the context of all trial groups receiving standard supportive care, two pragmatic comparisons will be made in evaluating MET for the facilitation of ureteric stone passage:

• Medical expulsive therapy (an α-blocker (tamsulosin) or a calcium-channel blocker (nifedipine)) versus placebo.

• An α-blocker (tamsulosin) versus a calcium-channel blocker (nifedipine).

The hypotheses being tested are:

1. The use of MET will result in an absolute increase in the spontaneous stone passage rate of at least 25% compared with placebo.

2. The use of an α-blocker (tamsulosin) will result in an absolute increase of 10% in the spontaneous stone passage rate compared with a calcium-channel blocker (nifedipine).

## Methods/design

**Figure 1:**
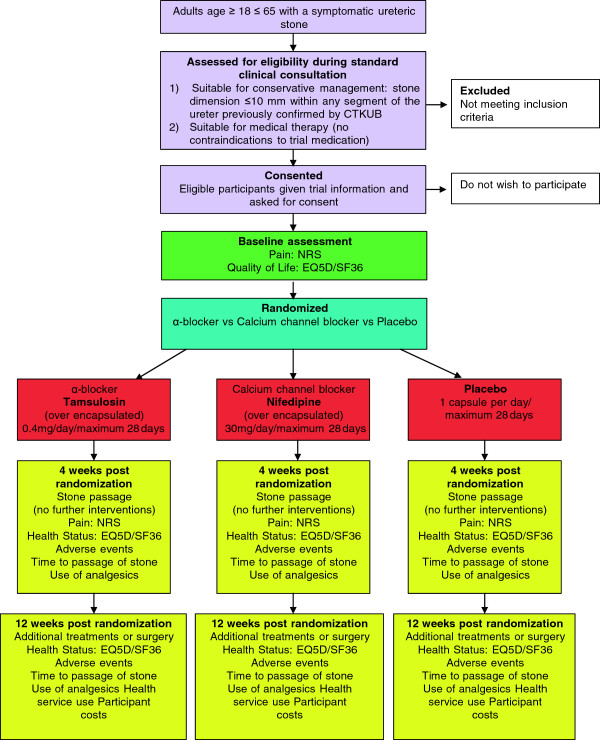
**Flow diagram of the SUSPEND trial.** CTKUB, Clinical tomography of the kidney, ureter and bladder; EQ5D, European Quality of Life, 5 Dimensions; NRS, numeric rating scale; SF36, Short Form (36) Health Survey.

This is a multicentre, double-blind, placebo-controlled, randomized trial evaluating two MET treatments (nifedipine or tamsulosin) versus placebo. The trial will be conducted in secondary care units with a high volume of admissions with ureteric stones across the UK. Figure 1 summarizes the trial design.

### Inclusion and exclusion criteria

#### Inclusion criteria

• Patients presenting acutely with ureteric colic.

• Adults between 18 and 65 years of age (inclusive).

• Presence of stone already confirmed by non-contrast computed tomography of the kidney, ureter and bladder (CTKUB).

• Stone within any segment of the ureter.

• Unilateral ureteric stone.

• Largest dimension of the stone ≤10 mm.

• Women must be willing to use two methods of contraception listed in the protocol prior to the start of dosing until at least 28 days after receiving the last dose of trial medication, or they must be postmenopausal (defined as 12 months with no menses without an alternative medical cause) or permanently sterilized.

• Capable of giving written informed consent, which includes compliance with the requirements of the trial.

#### Exclusion criteria

• Women who have a known or suspected pregnancy (confirmed by a pregnancy test).

• Women who are breastfeeding.

• Asymptomatic incidentally found ureteric stone.

• Stone not previously confirmed by CTKUB.

• Stone with any one dimension >10 mm.

• Kidney stone without the presence of ureteric stone.

• Multiple (that is ≥ 2) stones within ureter.

• Bilateral ureteric stones.

• Stone in a ureter draining a solitary kidney (either anatomically or functionally).

• Patients with abnormal renal tract anatomy (such as a duplex system, horseshoe kidney or ileal conduit).

• Presence of urinary sepsis.

• Chronic kidney disease stage 4 or stage 5 (estimated glomerular filtration rate < 30 ml/min).

• Patients currently taking an α-blocker.

• Patients currently taking a calcium-channel blocker.

• Patients currently taking PDE5 inhibitors.

• Contraindication or allergy to tamsulosin or nifedipine.

• Patients who are unable to understand or complete trial documentation.

Women who are eligible to take part in the trial and are of childbearing potential (that is, are not postmenopausal (defined as 12 months with no menses without an alternative medical cause) or permanently sterilized) will be advised to use two forms of highly effective birth control (that is, results in a less than 1% per year failure rate) and continue use until at least 28 days after the last dose of trial medication. Acceptable forms of contraception include:

• Established use of oral, transdermal, injected or implanted hormonal methods of contraception.

• Placement of an intrauterine device or intrauterine system.

• Barrier methods of contraception: condom or occlusive cap (diaphragm or cervical or vault caps) in combination with a spermicidal foam, gel, film, cream or suppository.

• The woman’s sole male partner is sterile (with the appropriate post-vasectomy documentation of the absence of sperm in the ejaculate) before her participation in the trial.

• True abstinence, when this is in line with the preferred and usual lifestyle of the subject. Periodic abstinence (for example, calendar, ovulation, symptothermal, postovulation methods) and withdrawal are not acceptable methods of contraception for trial purposes.

### Trial interventions

Two active treatments will be investigated:

1. Tamsulosin, 0.4 mg/day up to a maximum of 28 days.

2. Nifedipine, 30 mg/day up to a maximum of 28 days.

### Identification and enrolment of potential participants

As standard practice, clinicians will assess patients presenting with suspected ureteric calculi. A log will be taken of all patients assessed, in order to document the reasons for non-inclusion in the study (for example, reason they were ineligible, declined to participate) to inform the CONSORT diagram. Following adequate pain relief and confirmation of ureteric calculi by CTKUB, eligible patients (according to the inclusion and exclusion criteria) will be provided with a patient information leaflet. The information leaflet will be given to each potential participant to explain the benefits and known drawbacks of all aspects of this trial. It shall specifically explain that the trial will investigate the effect of two different drugs against a placebo, and explain the likelihood of participants receiving each of the three trial treatments. A member of the local research team will identify whether the patient is interested in the trial and will ensure that any questions the patients have are answered appropriately. The patients will be given as long as they require, prior to discharge, to make a decision about whether or not to participate. Signed informed consent forms will be obtained from the participants in all centres, by an individual who is appropriately trained. On providing consent, the patient will be asked to complete a baseline questionnaire and will then be randomized to one of the three treatment groups. The participant’s permission will be sought to inform their general practitioner (GP) that they are taking part in this trial.

### Randomization and allocation

Eligible and consenting participants will be randomized to one of the two intervention groups or the placebo group on a 1:1:1 basis using the proven telephone interactive voice response randomization application hosted by the Centre for Healthcare Randomized Trials (CHaRT) and the Health Services Research Unit in Aberdeen. The randomization algorithm will use centre, stone size and stone location as minimization covariates. Upon randomization, the participant will be allocated a unique participant study number and assigned a numbered participant pack containing over-encapsulated trial medication to ensure that the participant, investigator and trial personnel remain blind to treatment.

### Code break or emergency unblinding

Participants will be given a patient card, which will have the study title, investigational medicinal product details, participant ID and out-of-hours contact details, in case of emergency unblinding. The treatment code should only be broken for valid medical or safety reasons, for example, in the case of a severe adverse event, where it is necessary for the principal investigator or treating healthcare professional to know which treatment the participant is receiving, to determine emergency treatment. Where possible, the members of the research team should remain blinded, subject always to clinical need.

In the event of a clinical emergency requiring unblinding, the first point of contact is the local research team where the participant was recruited to the trial. The participant will be given a card to carry with details of a contact telephone number at the site (for example, the number for the on-call urologist), to be used in the event that unblinding is necessary. Contact information will also be available in the participant’s hospital notes.

A member of the research team or a member of staff at the local recruiting site will telephone the CHaRT randomization service at the number provided using the trial centre ID and the participant study number (the trial ID number will be available from the patient card or the participant’s hospital notes). The name and position of the person making the call will be recorded. In the unlikely event of randomization service failure, the on-call pharmacist at Aberdeen Royal Infirmary will be contacted and the same procedure followed.

Following any unblinding via the telephone randomization service, automatic emails will be sent to the chief investigator, trial manager and members of the CHaRT Management team. Similarly, the on-call pharmacist will email the same list of people to inform them of any unblinding. These emails will not contain the treatment code and the trial team will remain blinded as far as is practicable.

The chief investigator will then ascertain why unblinding has taken place. If the participant was unblinded because of a serious adverse event, an SAE form will be completed and will be reported as stipulated below (Safety – Procedure for recording adverse events).

### Trial procedures

Participants will complete three questionnaires. The baseline questionnaire will be completed in hospital after entry into the trial (but before participants receive their study medication). Two further postal questionnaires, one at 4 and one at 12 weeks post randomization, will be sent from the co-ordinating office (CHaRT, Aberdeen). In addition, participants may be reviewed in clinic at approximately 4 weeks after randomization (as is common in routine NHS care). In the event that the participant does not return the questionnaire, a reminder letter will be sent out approximately 2 weeks later.

Three case report forms will be completed by the research team at the recruiting site, one at baseline and one at the follow-up visit (in the case of non-attendance, this form will completed from the participant’s notes). If participants indicate on the 12-week questionnaire that they have had further interventions since their 4-week questionnaire, a further case report form will be completed from the participant’s notes. Additionally if the participant fails to return the 12-week questionnaire a 12-week CRF will be completed from their medical records.

**Figure 2:**
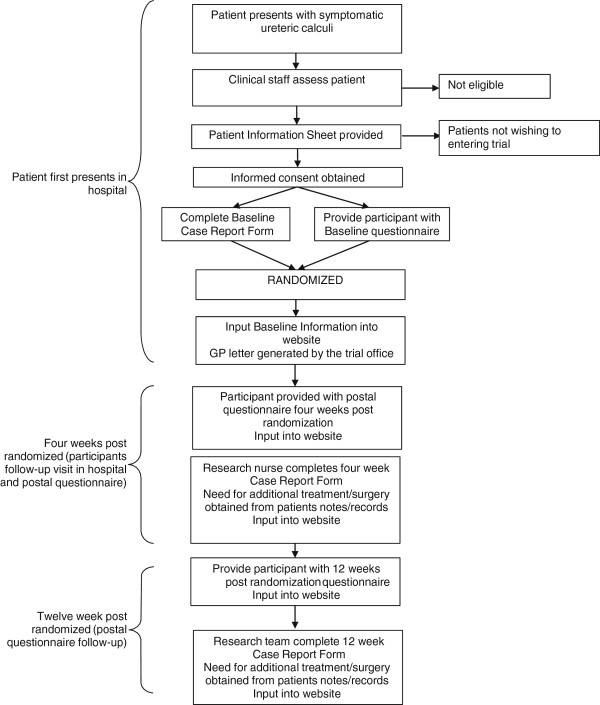
SUSPEND trial processes.

The complete trial processes are shown in Figure 2.

#### Subject withdrawal

Participants will remain on the trial unless they chose to withdraw consent or the principle investigator, chief investigator or trial manager feels it is no longer appropriate for the participant to continue (for example, the participant becomes unable to complete the trial documentation). Once the participant has been withdrawn from the trial, participant questionnaires will not be collected; however, permission will be sought for the research team to continue to collect outcome data from the participant’s hospital notes (using the case report forms).

In the event that participants are withdrawn from the study medication for any reason (for example, a serious adverse reaction or event occurs), they will still continue in the trial and will be asked to complete the trial documents.

### Trial medications

#### Investigational medicinal products

Tamsulosin hydrochloride (Petyme) will be sourced by Tayside Pharmaceuticals from Teva UK in the form of 400 μg modified release capsules.

Nifedipine (Coracten) will be sourced by Tayside Pharmaceuticals from UCB Pharma in the form of 30 mg sustained release capsules.

The placebo will be manufactured by Tayside Pharmaceuticals.

The medicinal products will be over-encapsulated to maintain the blinding of the trial. Trial medication will be presented as capsules in amber glass containers with a childproof closure and labelled according to Annex 13 of Volume 4 of *The Rule Governing Medicinal Products in the EU: Good Manufacturing Practices*[21].

The medicinal products and the placebo will be over-encapsulated, packaged and labelled by Tayside Pharmaceuticals according to good manufacturing practice.

#### Dosing regimen

Participants will take one capsule of the trial medication per day until stone passage occurs or for a maximum of 28 days.

#### Drug accountability

Blinded treatment packs will be stored by the local pharmacy according to manufacturer’s instructions until dispensed to the participant. Detailed dispensing records will be kept by the pharmacy.

#### Subject compliance

Upon randomization, each participant will be assigned a unique participant numbered pack of blinded medication. Participants will be instructed to store the medication according to the manufacturer’s instructions. Participants will be asked to record whether they completed the full course of treatment in the 4-week questionnaire. Unused medication or empty packaging should be returned by the participant at the 4-week follow-up visit and returned to the pharmacy. Participants who do not attend the 4-week visit will be instructed to destroy the medication. Participants will also be treated with the usual standard of care at the treating establishment, including prescribed analgesia.

#### Concomitant medications

Patients currently taking rifampicin or digoxin are excluded from the trial. Potential interactions are taken from the summary of product characteristics for each product; the participant’s GP will also receive this information.

### Outcome measures

The trial has a primary clinical and a primary economic outcome, reflecting the multidimensional nature of the possible effects the intervention may have.

#### Primary

• Clinical: the primary outcome is spontaneous passage of ureteric stones at 4 weeks (defined as no further intervention required to facilitate stone passage).

• Economic: a reduction in incremental cost per quality-adjusted life years (QALYs) gained at 12 weeks. The calculation of QALYs will be based on the participants’ responses to the EQ5D.

#### Secondary

• Patient-reported: a reduction in the severity of pain as measured by a numeric rating scale [22,23]. Generic health profile will be measured by the SF 36 and the use of analgesia.

• Clinical: time to passage of stone; further interventions received at 12 weeks.

• Safety: participant-reported discontinuation of trial medications.

• Economic: NHS primary and secondary care use and costs up to three months, incremental cost per surgical interventions averted; modelled incremental cost per QALY beyond the 12-week trial follow-up.

#### Timing and measurement of outcome assessment

Outcomes will be assessed at 4 weeks and 12 weeks post randomization, as shown in Table 1.

**Table 1:** Source and timing of measures

**Outcome measures**	**Source**	**Timing**		
		**Recruitment**	**Post randomization**	
			**4 weeks**	**12 weeks**
Need for additional intervention	Case report form		×	×
Additional interventions received	Participant questionnaire and case report form		×	×
Pain (numeric rating scale)	Participant questionnaire	×	×	
Health profile and status (SF36, EQ5D)	Participant questionnaire	×	×	×
Use of analgesics	Participant questionnaire		×	
Adverse events	Participant questionnaire		×	×
Time to passage of stone	Participant questionnaire and case report form		×	×
NHS primary and secondary healthcare use	Participant questionnaire and case report form			×
Participant out of pocket costs	Participant questionnaire			×

EQ5D, European Quality of Life, 5 Dimensions; SF36, Short Form (36) Health Survey.

### Safety

#### Timing and recording of safety parameters

Information regarding participant discontinuation of medication due to adverse events will be collected at 4 weeks in the participant questionnaire.

#### Procedures for recording and reporting adverse events

##### Adverse events that will not be reported

Non-serious events will not be collected or reported. Planned hospital visits for conditions other than those associated with the ureteric stone will not be collected or reported. Hospital admissions (planned or unplanned) associated with the treatment of the ureteric stone diagnosed at the time of entry to the trial are expected. These will be recorded as an outcome measure, but will not be recorded or reported as serious adverse events.

##### Procedure for adverse event recording in this trial

All adverse events will be assessed in respect of severity (serious or not), relationship to trial medication (suspected or not suspected), whether expected or unexpected, duration and, therefore, whether constituting a serious adverse event, by the local principle investigator or chief investigator, or their deputies.

Participants will be advised to contact their GPs should they experience an adverse event between the period following treatment and the 12-week follow-up questionnaire. This is current clinical practice for participants receiving tamsulosin or nifedipine within the NHS. When notified of trial participation, GPs will be asked to notify the trial office of any serious adverse reactions or events (for example, unexpected admission to hospital) in a timely manner. This will provide a robust system for the notification of any serious adverse reactions or serious adverse events (both expected and unexpected) occurring outside the trial visit.

1. Non-serious adverse events will not be collected. Participants will be asked whether they discontinued the study medication due to adverse reactions in the 4-week questionnaire.

2. All confirmed serious adverse events are to be notified to the co-sponsors and chief investigator as soon as the investigator or trial office (via a GP) becomes aware, either orally or in writing, of the event, and this should be followed by a written report on the event.

### Adverse reaction and unexpected adverse reaction reporting

#### Fatal or life-threatening suspected unexpected serious adverse reactions (SUSARs)

The chief investigator (or his designee) or co-sponsor will forward reports on fatal or life-threatening suspected unexpected serious adverse reactions (SUSARs) (that is, serious unexpected adverse events where a causal link between the drug and the event is suspected) to the Medicines and Healthcare Regulatory Authority (MHRA) and the research ethics committee within 7 days of their first knowledge of the minimum criteria using the relevant proforma. A copy will also be sent to the co-sponsor, the manufacturer and the data-monitoring committee. Follow-up information will be forwarded to the MHRA and ethics committee within 8 days.

#### Non-fatal and non-life-threatening SUSARs

The chief investigator (or designee) or co-sponsor will forward reports on non-fatal and non-life-threatening SUSARs to the MHRA and the research ethics committee within 15 days of their first knowledge of the minimum criteria using the relevant proforma. A copy will be sent to the co-sponsors, the manufacturer and the data-monitoring committee. Follow-up information will be forwarded to the MHRA and ethics committee within 8 days.

#### All other serious adverse reactions

The trial office, with the assistance of the chief investigator, will prepare a summary of all serious adverse reactions every six months. These will be distributed to the participating investigators, co-sponsors, manufacturer, trial steering committee and data-monitoring committee.

In addition, all suspected serious adverse reactions will be collated annually and submitted to theresearch ethics committee and the MHRA, in accordance with the guidance on annual safety reporting.

The data-monitoring committee will regularly assess the safety data collected for the trial and will have the ability to advise that the trial is temporarily or permanently halted based on safety concerns, according to the criteria defined in their charter.

#### Pregnancies

Pregnancy is not regarded as a serious adverse event, but will be recorded and reported. Pregnancy will be prevented as far as is practicable, but in the event a woman does become pregnant on the trial, she will be followed throughout her pregnancy and any serious adverse events at delivery will be recorded and reported. If necessary, the development of the newborn will be monitored for an appropriate period after delivery.

#### Sample size

The combined data from the two recent meta-analyses [17,18] suggest a relative risk of approximately 1.50, comparing MET (either α-blocker or calcium-channel blocker) against ‘standard care’ on the primary outcome. These reviews indicate a spontaneous stone passage rate of approximately 50% in control groups in the included randomized controlled trials. Only three of the included randomized controlled trials directly compared a calcium-channel blocker with an α-blocker, and these suggested that α-blockers are likely to be superior to calcium-channel blockers. Combining information from Singh [18] and Hollingsworth [17], stone passage rates for the α-blocker and calcium-channel blocker groups were approximately 85% and 75%, respectively. The most conservative sample size is required to detect superiority between the two active treatments and to this end will power the trial. To detect an increase of 10% in the primary outcome (spontaneous stone passage) from 75% in the calcium-channel blocker group to 85% in the α-blocker group, with type I error rate of 5% and 90% power, requires 354 per group; adjusting for 10% loss to follow-up inflates this to 400 per group. Since all treatment comparisons are pre-specified, no adjustment for multiplicity has been made [24]. Recruiting 1,200 participants (randomizing 400 to each of the three treatment groups; α-blocker, calcium-channel blocker and placebo) would provide sufficient power (>90%) for all other comparisons of interest.

#### Procedures to minimize bias

The trial will be conducted double blind, that is, the participant, investigator and personnel involved in the trial (with the exception of the data-monitoring committee members and allocated statistician) will be unaware of each individual’s treatment allocation.

#### Statistical analysis

Two comparisons will be considered for the primary trial analysis:

1. Medical expulsive therapy (an α-blocker (tamsulosin) or a calcium-channel blocker (nifedipine)) versus placebo.

2. An α-blocker (tamsulosin) versus a calcium-channel blocker (nifedipine).

Treatment groups will be described at baseline and follow-up using means (with standard deviations), medians (with interquartile ranges) and numbers (with percentages) where relevant. Primary and secondary outcomes will be compared using generalized linear models, with adjustment for participant baseline and minimization covariates: trial centre; stone size (≤5 mm or >5 mm to 10 mm); and location in ureter (upper, middle or lower). All estimates of treatment effect will be presented with 95% confidence intervals. Statistical significance will be at the 5% level. Primary analyses will be by allocated group (intention to treat). Subgroup analyses (appropriately analyzed by testing treatment by subgroup interaction) will explore the possible effect modification of a number of factors: stone size (≤5 mm or >5 mm to 10 mm); location in ureter (upper, middle or lower); and sex; all using stricter levels of statistical significance (*P* < 0.01, 99% confidence intervals).

All statistical analyses and reporting will follow a carefully documented statistical analysis plan. The trial steering committee and an independent data-monitoring committee will be asked to review and comment on the statistical analysis plan prior to any analysis and the plan will be finalized prior to any unblinding of the data. A single main analysis will be performed at the end of the trial when all follow-up has been completed. The data-monitoring committee will meet once 300, 600 and 900 participants have been randomized, to discuss interim analysis reports and the criteria for stopping the trial. The statistical analysis plan and data-monitoring committee charter will document the agreed timings and strategy.

### Economic evaluation

Economic evaluation will be an integral part of the trial. Resource use and costs will be estimated for each participant. Resource data collected will include the costs of the intervention drugs and simultaneous and consequent use of primary and secondary NHS services by participants. Personal costs, such as purchase of medications, time and travel will also be estimated. The perspective of the trial will be societal as it will include costs to both the NHS and the participants.

#### Collection of data

At recruitment, data will be collected on the intervention that the participants receive. Participants will be asked to provide information about their use of analgesics at 4 and 12 weeks post randomization, and about their primary and secondary healthcare service use at 12 weeks. They will also be asked for information about the time they spent travelling to primary and secondary health service providers and resources they might have used, such as mode of transport.

#### Participant costs

Participant costs will comprise self-purchased healthcare and travel and time costs for accessing NHS care. Self-purchased healthcare will include such items as prescription costs and over-the-counter medications. Information about these will be collected using a healthcare utilization questionnaire at 12 weeks. Participants will be asked for information on travel costs and this will be estimated from the number of visits to, for example, a GP or hospital doctor (estimated from the healthcare utilization questionnaire) and the unit cost of making a return journey to each type of healthcare provider (from the healthcare unit cost questionnaire).

The cost of participant’s time will be estimated by asking them how long they spent travelling to, and attending, their last visit to each type of healthcare provider. Participants will also be asked what activity they would have been undertaking (for example, paid work, leisure, housework) had they not attended the healthcare provider. This information will be presented in natural units, for example, hours, and also costed using standard economic conventions [25-27]. These unit time costs, measured in terms of their natural and monetary terms, will then be combined with estimates of number of healthcare contacts derived as outlined next.

#### NHS costs of other health services used

Use of secondary care services following the treatment period will be collected using a case report form. This form will record information on non-protocol outpatient visits (protocol visits are those scheduled for the purposes of data collection) and readmissions relating to the use and consequences of drug treatment. Data on the use of primary care services, such as prescription medications, contacts with primary care practitioners, for example, GPs and practice nurses, will be collected via the healthcare utilization questionnaire completed at the 12-week follow-up.

#### Cost-effectiveness

The cost-effectiveness within the trial will be measured in terms of the number of participants needing further treatment during or at the end of the 12-week follow-up and the number of QALYs at 12 weeks. The number of QALYs will be estimated by combining estimated quantity of life, with quality of life derived from the EQ5D questionnaire (administered at baseline, 4 weeks and 12 weeks post randomization) and UK tariffs [28].

The within-trial analysis will be based on the 12-week follow-up and the results will be presented as the incremental cost per further treatments needed during or at the end of the 12-week follow-up and the incremental cost per QALY gained. The results will be presented as point estimates of mean incremental costs, number of further treatments needed, QALYs, incremental cost per further treatment needed and incremental cost per QALY. Measures of variance for these outcomes are likely to involve bootstrapping estimates of costs, number of further treatments needed, QALYs, incremental cost per further treatments needed and incremental cost per QALY. Incremental cost-effectiveness data will be presented in terms of cost-effectiveness acceptability curves. Forms of uncertainty, for example, concerning the unit cost of a resource from the different centres, will be addressed using deterministic sensitivity analysis. Where feasible, the results of the sensitivity analyses will also be presented as cost-effectiveness acceptability curves.

A modelling exercise will be performed to extrapolate the estimates of the cost-utility analysis to a longer time horizon than that considered by the trial. This will allow consideration of the costs of any subsequent treatments performed after the trial follow-up period and effects on quality of life prior to this. Consideration will also be given to the relevance of costs and effects on quality of life following subsequent treatment. Individual participant data from the trial as well as both published and unpublished evidence in the field will be used to populate the model. The methods used to assemble additional data will follow recognized methodologies, which will vary according to the type of parameter, extent of uncertainty and role within the model. Therefore, comprehensive systematic searching will be limited to those parameters to which the results of the model are likely to be particularly sensitive. The modelling exercise will comply with recent recommendations on good practice for modelling; results will be presented in terms of incremental cost per additional treatment needed and incremental cost per QALY gained. Parameters and other forms of uncertainty will be addressed using probabilistic and deterministic sensitivity analysis.

### Ethical approval

The East of Scotland Research Ethics Committee reviewed and approved this study on 15 June 2010 (REC reference number, 10/S0501/31). The trial is conducted in accordance with the principles of Good Clinical Practice and complies with the Declaration of Helsinki (2000).

## Discussion

The main practical challenge of this trial has been the identification and recruitment of participants, owing to the nature of ureteric stone disease and the care pathway for these patients within the NHS. The majority of patients present to secondary care units via emergency departments, often out of normal hours, and this is problematic for site research staff, as there is no way of predicting how, where and when these patients might seek treatment. The availability of the clinical trial pharmacist (often only during normal office hours) to dispense trial medication has also had implications for recruitment. In some cases, participants have volunteered to come back and collect trial medication during pharmacy hours, but there have been occasions where they have not complied with this requirement.

As the trial has progressed, we have seen a lower than expected response rate to the participant questionnaires. Strategies to improve response rate have been employed, including text-message pre-notification of questionnaire delivery, email delivery of reminder questionnaires, short- form questionnaires at 12 weeks (capturing the EQ5D data only) and monetary incentives (unconditional £5 gift voucher for UK high street stores) sent with the 12-week questionnaires. The impact of these will be discussed in the full trial report.

Our choice of primary outcome measure, where stone passage is defined as no further intervention required, is a pragmatic one and encompasses patient (symptoms), clinical (continued presence of stone), and healthcare delivery aspects of stone treatment. Although previous triallists have recorded stone-free rate as confirmed by imaging techniques (for example, CTKUB, plain X-ray of the kidney, ureter and bladder), we considered that participants would not receive imaging as part of their standard care in the NHS and that there are safety concerns with asking them to undergo such procedures purely to fulfil the requirements of the trial. Additionally, stone-free rate confirmed by imaging does not allow for a cost-effectiveness assessment, which will be key to evaluating the effect of MET. Any possible centre and other biases relating to outcome attribution and recording should be mitigated by blinding of the participants and healthcare professionals, randomization and the high sample size of the trial.

Other than these problems, there have been no main issues in conducting the SUSPEND trial. We advise researchers considering studies in a similar setting to consider carefully the practicalities of recruitment and availability of research staff; both those involved in the trial and those in supporting services (for example, the clinical trial pharmacy).

## Trial status

The first participant was recruited in January 2011 and the trial is currently open to recruitment in 25 UK centres.

## Abbreviations

CHaRT: Centre for Healthcare and Randomised Trials; CONSORT: Consolidated Standards of Reporting Trials; CTKUB: clinical tomography of the kidney, ureter and bladder; EQ5D: European Quality of Life, 5 Dimensions; GP: general practitioner; MET: medical expulsive therapy; MHRA: Medicines and Healthcare Regulatory Authority; NHS: National Health Service; QALY: quality-adjusted life year; SAE: serious adverse event; SF36: Short Form (36) Health Survey; SUSAR: suspected unexpected serious adverse reaction; SUSPEND: Spontaneous Urinary Stone Passage ENabled by Drugs.

## Competing interests

The authors declare that they have no competing interests.

## Authors’ contributions

SMcC (chief investigator) and GMcL: conception and design, data collection and analysis, manuscript writing and final approval of the manuscript. KS: design, data collection and analysis, manuscript writing and final approval of the manuscript. RT, JND, RP, KA and JB: conception and design, manuscript writing and final approval of the manuscript. GMcP and AMcD: data collection, manuscript writing and final approval of the manuscript. TL and MK: design, manuscript writing and final approval of the manuscript. All authors read and approved the final manuscript.
